# Oil-in-Water Nanoemulsion Can Modulate the Fermentation, Fatty Acid Accumulation, and the Microbial Population in Rumen Batch Cultures

**DOI:** 10.3390/molecules28010358

**Published:** 2023-01-01

**Authors:** Mohamed El-Sherbiny, Mostafa S. A. Khattab, Ahmed M. Abd El Tawab, Mostafa Elnahr, Adam Cieślak, Małgorzata Szumacher-Strabel

**Affiliations:** 1 Department of Dairy Science, National Research Centre, 33 Bohouth St., Dokki, Giza 12622, Egypt; 2 Animal Production Department, Faculty of Agriculture, Al-Azhar University, Cairo 11884, Egypt; 3 Department of Animal Nutrition, Faculty of Veterinary Medicine and Animal Science, Poznań University of Life Sciences, Wołyńska 28, 60-637 Poznań, Poland

**Keywords:** rumen, fermentation, nanoemulsion, oil, fatty acids, microbial population

## Abstract

In this study, three oil-in-water nanoemulsions were tested in two stages: In the first stage, three levels (on the substrate dry matter (DM)), namely 3%, 6%, and 9%, of three different oils, olive oil (OO), corn oil (CO), and linseed oil (LO), in raw and nanoemulsified (N) forms were used separately in three consecutive rumen batch cultures trials. The second stage, which was based on the first stage’s results, consisted of a batch culture trial that compared the raw and nanoemulsified (N) forms of all three oils together, provided at 3% of the DM. In the first stage, NOO, NCO, and NLO preserved higher unsaturated fatty acid (UFA) and less saturated fatty acid (SFA) compared to OO, CO, and LO, respectively; noticeably, NCO had UFA:SFA = 1.01, 1.16, and 1.34 compared to CO, which had UFA:SFA = 0.66, 0.69, and 0.72 when supplemented at 3%, 6%, 9% of DM, respectively. In the second stage, UFA:SFA = 1.04, 1.12, and 1.07 for NOO, NCO, NLO, as compared to UFA:SFA = 0.69, 0.68, and 0.72 for OO, CO, and LO supplemented at 3% of DM. In conclusion, oil-in-water nanoemulsions showed an ability to decrease the transformation of UFA to SFA in the biohydrogenation environment without affecting the rumen microorganisms.

## 1. Introduction

Over several decades, numerous feed supplements have been introduced to moderate the proportions of fatty acids found in the rumen and, consequently, modify ruminant products’ fatty acid content [1]. There is a significant interest in the research and development of nutritional strategies to increase milk and meat’s unsaturated fatty acids (UFAs) content [1,2]. The conjugated linoleic acid (CLA) isomers and omega-3 and omega-6 fatty acids are primarily responsible for the potential health benefits associated with UFA consumption [2]. Increasing the amount of unsaturated fatty acids (UFAs) in the daily human diet can help avoid inflammatory conditions and prevent or delay the development of atherosclerosis and coronary heart disease [2]. According to O’Donnell [3], fat from dairy products contributes approximately 25–35% of the total daily intake of saturated fatty acids and up to 15–25% of the total daily fat intake. According to the same literature, approximately 70% of the fatty acids in the milk fat of ruminants are saturated fatty acids (SFAs), 8% are trans fatty acids, and less than 5% are long-chain unsaturated fatty acids (LCUFAs) [3]. The consumption of saturated fatty acids is associated with an increased risk of cardiovascular disease, obesity, and atherosclerosis [4,5]. On the other hand, LCUFAs, particularly Omega 3, Omega 6, and conjugated linoleic acid (CLA), are well-known for their significance as active dietary compounds for preventing arteriosclerosis, coronary heart disease, and inflammatory conditions and inhibiting the growth of tumor cells, according to the results of studies conducted on various animal models (including mice, rats, and pigs) [2,5]. Omega-3 is also essential for infant brain, eye, and nervous system development and has been shown to promote long-term heart health, as supported by trials on mice and rats and clinical studies on children [2,6]. Edible oils are among the forms of UFA supplementation utilized to alter the fatty acid composition of ruminant products. Oils can modify the rumen fatty acid proportion by affecting the activity of rumen microorganisms, particularly the bacterial population, mainly biohydrogenation bacteria such as *Butyrivibrio fibrisolvens* and *Butyrivibrio proteoclasticus*, which have been shown to change the fatty acid content of the rumen by specifically affecting the accumulation of long-chain fatty acid and the formation of trans-vaccenic acid and conjugated linoleic acid [7,8]. However, some constraints and limitations are placed on the use of edible oil supplementation in ruminant nutrition due to the potential to hurt rumen fermentation [8]. During the fermentation process in the rumen, dietary lipids cause changes in the digestion pattern, some of which could lead to a reduction in the composition of volatile fatty acids (VFAs) [9]. It is also known that the rumen produces less methane when there is more fat in the diet because dietary fat decreases the amount of hydrogen that is accumulated through the process of fatty acid biohydrogenation, the amount of fermentable organic matter that is consumed, the rate of fiber digestion, and the count and activity of the ruminal bacteria [10]. Because of these limits, it is desirable to find other dietary sources of UFA that can affect and modulate the proportion of fatty acids in the rumen without detrimental effects on the microorganisms or rumen fermentation indicators. Rumen microorganisms biotransform dietary unsaturated fatty acids into saturated fatty acids to protect themselves from the detrimental effect of UFA on their cell structure, particularly polyunsaturated fatty acids, which can disrupt the membrane integrity of the rumen biohydrogenation bacteria [7,8,9]. These processes ultimately increase the outflow of saturated fatty acids to post-ruminal digestion and absorption [9,10]. Therefore, it is desirable to investigate other forms of supplemented oils for the ruminant diet that could prevent the PUFAs from being digested by rumen lipolysis and biohydrogenation without significantly affecting the rumen fermentation pattern and the rumen microorganisms [11,12]. In ruminant nutrition, the oil-in-water nanoemulsion is a relatively new form; it is, however, one of the most important nanotechnologies with numerous scientific and practical applications. Nanoemulsions are multiphase colloidal dispersions formed by dispersing one liquid in another immiscible liquid by physical-share-induced rupturing at the nanoscale, with droplet sizes less than 100 nm [13]. Based on previous in vitro studies [14,15], a higher outflow of unsaturated fatty acids from the rumen (bypassing the rumen) may be anticipated when introducing a nanoemulsified form of edible oil to the rumen fermentation environment; as a result, higher accumulation of unsaturated fatty acids may be observed. Consequently, the hypothesis-driven idea is that the small size of dietary edible oil droplets (nanoscale) produced with nanoemulsion technology will have a smaller impact on the rumen fermentation and rumen microorganisms than raw oils. Considering this, the purpose and aim of the following study were to evaluate the effects of nanoemulsified olive oil, corn oil, and linseed oil on ruminal fermentation, particularly volatile fatty acid production, fatty acids accumulation, and microbial population, using in vitro batch fermentation culture trials.

## 2. Materials and Methods

**Figure 1:**
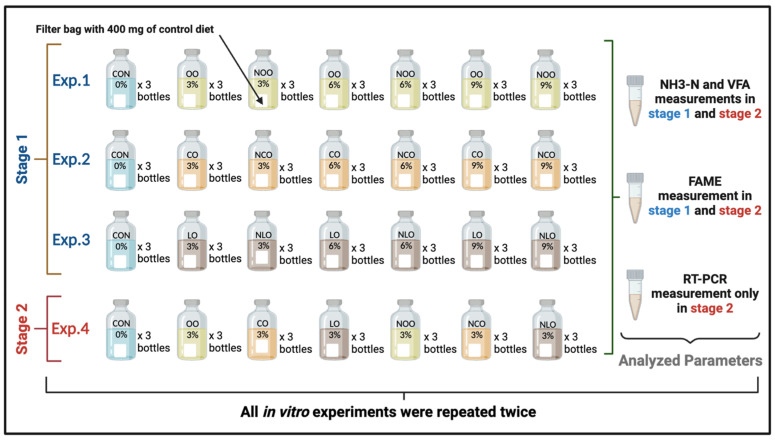
The experimental design and management. The treatments are as follows: CON, control diet; OO, raw olive oil; NOO, nanoemulsified olive oil; CO, raw corn oil; NCO, nanoemulsified corn oil; LO, raw linseed oil; and NLO, nanoemulsified linseed oil.

The experimental design and management are illustrated in Figure 1. Briefly, three different plant oils, namely olive oil (OO), corn oil (CO), and linseed oil (LO), in two forms, i.e., raw form (OO, CO, and LO) and nanoemulsified form (NOO, NCO, and NLO), were evaluated in two different stages: The first stage consisted of three consecutive in vitro rumen batch fermentation culture trials, testing each oil separately, supplemented at 3%, 6%, and 9% of the substrate dry matter. The second stage consisted of a fourth in vitro trial designed based on the promising results obtained from the first stage. The oils used were chosen based on an earlier physiochemical evaluation of nanoemulsions produced from eight different edible oils purchased from local markets in Egypt; the results showed a moderate fatty acid composition and better emulsion stability of the OO, CO, and LO nanoemulsions compared to nanoemulsions produced from sunflower oil, soybean oil, cottonseed oil, sesame seed oil, and fish oil. The in vitro trials and all related analyses of the collected samples throughout the studies were conducted at the laboratory of Dairy Production of the Department of Dairy Science, National Research Centre, Giza, Egypt.

### 2.1. Oil-in-Water Nanoemulsion Preparation and Evaluation

Edible oils in water were premixed at 13,500 rpm for 2 min with a digital high-speed homogenizer (HG-15D Homogenizer, Daihan Scientific C., Gangwon-Do, South Korea) to achieve a droplet size distribution of 6.1 ± 0.4 μm. Then, using the pre-homogenized solution, oil-in-water nanoemulsion was prepared by using a Sonics VCX750 ultrasonic processor of 750-Watt nominal power and frequency of 20 kHz, equipped with a 25 mm sonotrode tip (80% amplitude for 20 min; Sonics and Materials, Newtown, CT, USA) [16]. The oil-in-water emulsion consisted of 15% of edible oil (olive oil, corn oil, and linseed oil bought from Egyptian markets), 5.6% Tween 80 (Sigma Aldrich, Darmstadt, Germany), and 79.4% distilled water. Before each in vitro trial, collected samples of nanoemulsions were screened for droplet size and zeta potential; initially, samples were added to 3520C-MB SPI supplies carbon-coated 200 mesh copper grids with an additional staining agent, phosphotungstic acid of 1% concentration, and then screened with JEOL JEM-2100 high-resolution and analytical electron microscope equipped with STEM unit (bright- and dark-field detectors) and EDXS detector. Later, a Zetasizer Nano ZS was used to measure the nanoemulsion droplet size and zeta potential at 25 °C (Malvern Instruments, Malvern, United Kingdom).

### 2.2. The In Vitro Fermentation Trials

Four consecutive in vitro batch fermentation culture trials (Figure 1) were conducted to evaluate the effect of nanoemulsified olive oil (OO), corn oil (CO), and linseed oil (LO) on the rumen fermentation pattern, microbial population, and fatty acid formation and accumulation in the fermentation fluid. The in vitro trials were performed by following the batch culture incubation procedure stated in [14], but with a few modifications.

#### 2.2.1. Source of the Rumen Inoculum

The rumen inoculum was obtained from three cows from the El-Munib slaughterhouse, Giza, Egypt. Slaughtered cows had free access to fresh water and were fed ad libitum on a diet containing concentrates mixture and berseem hay at a ratio of 50:50 on the dry matter (DM) basis. The ruminal content was collected separately from each cow’s top, bottom, and middle sections of the rumen. The ruminal contents from all three cows were then equally blended and strained through 4 layers of cheesecloth into a Schott Duran bottle (Schott North America Inc., Elmsford, NY, USA), which was maintained at 39 °C under anaerobic conditions. The collected ruminal fluid was immediately transported to the laboratory of Dairy Production, National Research Centre, Giza, Egypt; placed in a water bath preheated to 39 °C; and mixed in a beaker before diluting with the buffer solution.

#### 2.2.2. Trials Design and Management

Table 1 lists the components and chemical composition of the control substrate (feed), while Table 2 lists the fatty acid composition of the control substrate and used supplements. The used substrate is identical to the control diet used later in an experiment on dairy goats [17]; all components of the control substrate were first dried, and each dried component was milled separately. Subsequently, a DM-based homogenous mixture of the experimental substrate was created by combining all the dry milled ingredients listed in Table 1. Two hours before the start of the experiment, a portion of 400 mg of the prepared dry substrate was weighed into filter bags (ANKOM F57; Ankom Technology, Macedon, NY, USA) and transferred to the corresponding glass incubation bottle; the bottles were then moved to the incubator set to 39 °C (prewarming the feed). The rumen fluid was diluted with a buffer solution at a ratio of 1:4 (292 mg of K_2_HPO_4_·3H_2_O, 240 mg of KH_2_PO_4_, 480 mg of (NH_4_)_2_SO_4_, 480 mg of NaCl, 100 mg of MgSO_4_·7H_2_O, 64 mg of CaCl_2_·2H_2_O, 4 mg of Na_2_CO_3_, and 600 mg of cysteine hydrochloride per liter). Then 40 mL of this mixture was added to the 125 mL glass incubation bottle containing the dried substrate in a filter bag. Before adding the buffered rumen fluid, the levels of raw oil (3%, 6%, and 9%) were calculated based on the DM of the substrate and added directly to the 400 mg substrate (above the filter bags). The oil content of the prepared nanoemulsified form was equivalent to the oil doses used in the treatment of crude oil. However, the amounts of the supplemented nanoemulsions (3%, 6%, and 9%) were recalculated based on the oil content (15%) of the nanoemulsion preparation to be added at approximately 20%, 40%, and 60% of the DM basis of the substrate. In contrast to the raw oil form, the nanoemulsified oil was added directly to the bottle containing the buffered rumen fluid and bags containing the substrate to simulate the drinking process, which was later applied to the experiment with lactating goats [17]. Three replicates of batch culture fermentation trials were conducted for the four in vitro experiments (three bottles were used for each treatment). Three bottles contained only the dry control substrate, and three additional bottles that had only culture fluid without the substrate (blank) were also used. Each in vitro experiment was repeated twice, and each run (repetition) began with a new rumen fluid collection. The bottles were filled with carbon dioxide, sealed with a rubber stopper, and tightened with an aluminum cover. The bottles were then incubated for 24 h in an anaerobic environment with a pH of 6.5, a temperature of 39 °C, and a 100 rpm shaking incubator.

#### 2.2.3. Substrate and Incubation Fluid Sampling

At the beginning of each experiment run, used substrate samples were collected and stored at −20 °C until undergoing chemical analysis in triplicate. After 24 h of incubation, the total gas production was determined for each trial repetition by subtracting the volume of gas produced in flasks containing substrate and buffered rumen fluid from the volume of gas produced in blank flasks. The bottles were then placed in a refrigerator, at 5 degrees Celsius, to stop the fermentation process. The filter bags were removed from the bottles and dried at 50 °C for 48 h in a forced-air oven. Three glass tubes containing 5 mL of fermentation fluid samples were collected from each bottle and analyzed for ammonia nitrogen (NH-N), volatile fatty acid (VFA), fatty acid methyl esters (FAME), and a selected microbial population.

### 2.3. Samples Chemical Analysis

#### 2.3.1. Control Substrate Basic Analysis

Before chemical analysis, concentrate and roughage samples collected during the study were dried at 55 °C for 48 h, milled to pass a 1 mm screen (FZ102, Shanghai-Hong Ji Instrument Co., Shanghai, China), and composited. Analytical DM (method no. 934.01), ash (method no. 942.05), crude protein (method no. 954.01), and ether extract (method no. 920.39) were measured on the collected samples [18]. Using an ANKOM200 Fiber Analyzer unit, neutral detergent fiber (NDF; [19]) and acid detergent fiber (ADF; AOAC [18]; method 973.18), analyses were conducted (ANKOM Technology Corporation, Macedon, NY, USA). Samples were pretreated with alpha-amylase and sodium sulfite before NDF analysis. NDF and ADF are expressed without ash residue, and organic matter (OM) was calculated by using the difference.

#### 2.3.2. Fermentation Characteristics

The pH was measured immediately in the batch fermentation culture trials before transferring the bottle to the refrigerator, using a benchtop pH meter (Orion star pH meter, Thermo Fisher Scientific Inc., Erlangen, Germany). For each trial repetition, the total gas production (TGP) was calculated by subtracting the volume of gas produced in flasks containing substrate and buffered rumen fluid from the volume of gas produced in flasks containing no substrate or rumen fluid. The in vitro dry matter degradations (IVDMDs) were determined by subtracting the initial (substrates) and final (residues) substrate DM weights. According to [15], the ammonia nitrogen (NH3-N) concentration was measured by using the colorimetric Nessler method. The volatile fatty acids (VFAs) were analyzed according to [15], but with minor modifications. Briefly, 0.8 mL of fermentation fluid and 0.2 mL of a solution containing 250 g of metaphosphoric acid/L were combined. Using a gas chromatograph (GC) with an automated sampler (Model 7890B; Agilent Technologies, Palo Alto, CA, USA), the concentration of VFA and its molar proportions were determined at the Central Laboratories Network, National Research Centre, Cairo, Egypt. The GC–MS was outfitted with a capillary column HP-FFAPv (19091F-112; 0.320 mm outer diameter, 0.50 m inner diameter, and 25 m length; J & W Agilent Technologies Inc., Palo Alto, California, United States). As an external standard (Sigma Chemie GmbH, Stein, Germany), a mixture of known concentrations of individual short-chain fatty acids (acetate, propionate, and butyrate) was used to calibrate the integrator. The VFA peaks were identified qualitatively and quantitatively, using external standards prepared by mixing Fluka-purchased individual VFA (Sigma Aldrich, MO, USA). MS Workstation 5.0 was utilized for data processing.

#### 2.3.3. Fatty Acid Methyl Esters Analysis

The fatty acid methyl ester (FAME) analysis procedure was outlined in [14] for the samples of fermentation fluid and dry ground feed; 2500 µL and 100 mg of fermentation fluid and dry feed samples, respectively, were added to 3 mL of 2 M NaOH for hydrolysis of the samples in a closed system employing 15 mL screw-cap Pyrex tubes with Teflon stoppers. The hydrolyzed samples were incubated in a block heater for 40 min at 90 °C. The extracted samples were then esterified in methanol with 0.5 M NaOH and converted to FAME in boron trifluoride (1.3 M; Fluka-Sigma Aldrich, St. Louis, MO, USA). Utilized as a gas GC–MS system (7890B, Agilent, Santa Clara, CA, USA) with a mass spectrometer detector and a 100 m fused-silica capillary column (0.25 mm i.d., coated with 0.25 m Agilent HP; Chrompack CP7420; Agilent Technologies, Santa Clara, CA, USA) (5977A). Throughout the FAME chromatographic analysis, the carrier gas was hydrogen, which flowed at a rate of 1.3 mL/min. The injector and detector temperatures were 200 °C and 250 °C, respectively. The temperature of the oven was set to begin at 120 °C for 7 min before increasing by 7 °C per minute to reach 140 °C, where it remained for 10 min before being increased by 4 °C per minute to 240 °C. Then 1 µL of the sample was injected into the GC column. The peaks were identified by comparing their retention times to those of the relevant FAME standards (37 FAME Mix, Sigma Aldrich, PA, USA), utilizing Open Lab CDS version 2.6. (Agilent, Santa Clara, CA, USA). In addition, the retention times of a reference standard and the conjugated linoleic acid peaks were compared to identify them (a mixture of cis- and trans-9,11 and 10,12-octadecadienoic acid methyl esters; Sigma Aldrich, PA, USA), the compositions of FA were given as g/100 g of total FA. The Central Service Unit at the National Research Centre in Egypt performed the chromatographic FA analyses.

#### 2.3.4. Rumen Bacteria Quantification

Using real-time PCR, eight rumen bacteria were quantified. According to [15], metagenomic DNA was extracted from rumen fluid, using a QIAamp DNA Stool mini kit (Qiagen GmbH, Hilden, Germany). Using the BLAST program in the GenBank database, the specificity of primers was verified (Table 3). The QuantStudio 12 Flex PCR system quantified specific bacteria with a known initial DNA concentration (25 ng/L) (Life Technologies, Thermo Fisher Scientific, Waltham, MA, USA). The Power SYBR Green PCR Master Mix was utilized for PCR amplification (Thermo Fisher Scientific, Waltham, MA, USA). The reaction mixture contained 4 L of the 2X master mix, 25 ng of template DNA, and 0.5 M of each primer in a final volume of 10 L. The following amplification protocol was utilized: one cycle of amplification at 95 °C for 10 min for initial denaturation, 45 cycles at 95 °C for 15 s, followed by annealing at temperatures (dependent on the analyzed bacteria) for 5 s, and finally at 62 °C for 67 s. In the final phase of each cycle, fluorescent by-products were discovered. An analysis of product melting after a single amplification (0.1 °C increments from 65 °C to 95 °C, with fluorescence collection at 0.1 °C intervals) was performed to explain the characteristics of amplification. Using the formula 2^−△△CT^ (RTA), the relative abundances of DNA copies of each bacterial species relative to the total bacteria were calculated.

### 2.4. Statistical Analysis

In the initial statistical analysis phase, 3-way ANOVA and the Tukey post hoc test were utilized. The primary effects of oil (nanoemulsified versus raw), supplementation level (0%, 3%, 6%, and 9%), experiment repetition, and the interaction between oil type and supplementation level were determined. However, we found no effect of experiment repetition on the obtained results; thus, it was eliminated from the model, and all data were re-analyzed by using two-way ANOVA. For all oil types, linear and quadratic responses to the level of supplementation were described by using polynomial contrasts. In the second phase, data were analyzed by using a one-way ANOVA with the treatment as a constant factor. All analyses were conducted by using SAS software (SAS^®^ OnDemand for Academics, 2022 SAS Institute Inc., Cary, NC, USA). At *p* ≤ 0.05 and 0.05 < *p* ≤ 0.10, respectively, treatment effects were deemed significant or trending toward significance. For each value, the means and pooled standard errors of the means are displayed.

## 3. Results

### 3.1. Oil-in-Water Nanoemulsion Evaluation

**Figure 2:**
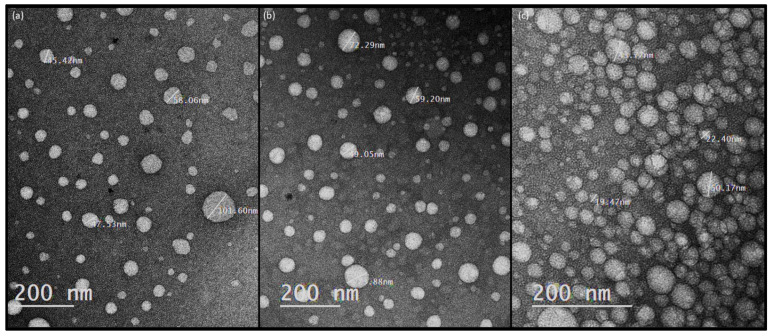
Transmission electron microscope micrographs, showing the droplet size of oil-in-water nanoemulsions prepared from three different oils: (**a**) olive oil-in-water nanoemulsion, (**b**) corn oil-in-water nanoemulsion, and (**c**) linseed oil-in-water nanoemulsion.

**Figure 3:**
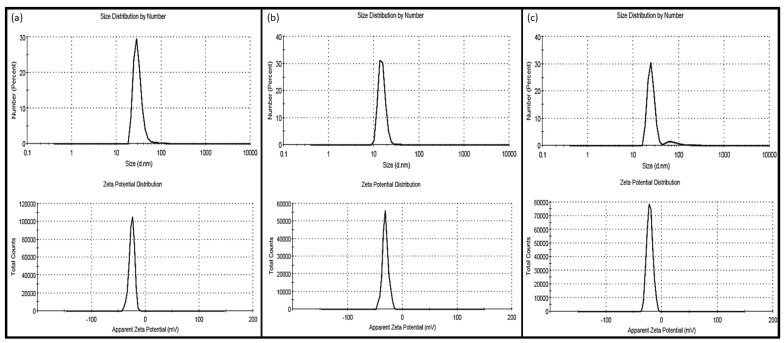
Size distribution (nm) and apparent Zeta potential (mV) of oil-in-water nanoemulsified samples of three different nanoemulsions: (**a**) olive oil-in-water nanoemulsion, (**b**) corn oil-in-water nanoemulsion, and (**c**) linseed oil-in-water nanoemulsion.

Some of the results of droplet size and zeta potential of the prepared nanoemulsions are shown in Figure 2 and Figure 3. In all scanned nanoemulsion samples, the droplet size distribution ranged from 30 to 180 nm, with 90% averaging 60 ± 8 nm. The zeta potential was also observed to be more negative than -−30 mV in all oil-in-water nanoemulsions, which is considered a stable emulsion. However, in our previous chemical and physical screening studies (unpublished data), we observed that the oil-in-water nanoemulsion is sensitive to storage time, showing a positive correlation starting from day 10 of storage, reaching a size distribution range of 10–58 µm at day 30 of storage, and the storage temperature showing a positive correlation, especially for a temperature less than 10 °C and higher than 60 °C, for 24 h increased the droplet size significantly.

### 3.2. First Stage of Trials

#### 3.2.1. Olive Oil Supplementation

Table 4 summarizes the effects of raw (OO) and nanoemulsified (NOO) olive oil supplementation on basic rumen parameters, volatile fatty acid, and fatty acid composition. The OO levels of supplementation had a negative impact on the pH level, total gas production (TGP), ammonia, and in vitro dry matter degradation of the fermentation culture (IVDMD). With the increasing OO supplementation, the pH of the fermentation fluid decreased linearly and quadratically (*p* < 0.01). Following the decrease in ammonia concentration, the IVDMD decreased quadratically (*p* < 0.001); the observed reduction in both ammonia concentration and IVDMD was most pronounced at the highest levels of OO addition (6% and 9%). For NOO, quadratic decreases (*p* < 0.001) in pH and IVDMD were only observed at the highest supplementation level (9%) (*p* < 0.001). The volatile fatty acid appears to be adversely affected by OO supplementation, particularly the decrease of (*p* 0.01) the acetate and propionate both linearly and quadratically as compared to NO, which has a less severe effect on the volatile fatty acid. Regarding fatty acid proportion, OO supplementation led to a quadratic increase (*p* < 0.01) in the proportion of trans-11 C18:1 (vaccenic acid, VA). In contrast, the proportion of VA decreased quadratically (*p* < 0.01) as the NOO addition increased. Similarly, the proportion of cis-9 trans-11 C18:2 tended to increase linearly (*p* < 0.05) with OO supplementation. Regarding C18 unsaturated fatty acids, as OO levels increased, the proportions of oleic acid (OL) and linoleic acid (LA) increased quadratically (*p* < 0.01 and *p* < 0.05, respectively). The increase in OO levels quadratically decreased (*p* < 0.01) the sum of saturated fatty acids (SFA) and led to a quadratic increase (*p* < 0.05) in the sum of polyunsaturated fatty acids (PUFA). Although OO supplementation linearly decreased SFA and increased PUFA, changes in proportions were still less than those obtained with the NOO addition, which reduced PUFA to a lesser extent, while demonstrating a quadratic increase (*p* < 0.01) in PUFA relative to the proportion of SFA, which tended to decrease quadratically (*p* < 0.01) with nanoemulsified olive oil supplementation.

#### 3.2.2. Corn Oil Supplementation

The effects of raw (CO) and nanoemulsified (NCO) corn oil supplementation on basic rumen parameters, volatile fatty acid, and fatty acid composition are summarized in Table 5. Similar to OO, the addition of CO had a negative effect on the pH, total gas production (TGP), ammonia, and in vitro dry-matter degradation of the fermentation culture (IVDMD). It was observed that the addition of CO led to a quadratic decrease in pH (*p* < 0.01). This decrease in pH was associated with a quadratic (*p* < 0.01) decrease in ammonia and IVDMD. For the NCO, the obtained results were identical to those for the NOO, in which an increase in the NCO addition led to a linear decrease (*p* < 0.01) in the pH, ammonia, and IVDMD in the fermentation culture. Regarding the VA and cis-9 trans-11 C18:2 proportions, CO and NCO supplementation led to a similar pattern of results to that observed when OO and NOO were added. We observed a quadratic increase in vaccenic acid (*p* < 0.01) with increasing levels of CO supplementation and a linear increase in cis-9 trans-11 C18:2 proportions (*p* < 0.01) with the addition of CO. The nanoemulsified supplementation significantly decreased (*p* < 0.05) the proportions of VA, CLA isomer, and PUFA.

#### 3.2.3. Linseed Oil Supplementation

In Table 6, NLO levels up to 9% led to a quadratic elevation in the C18:1 cis-9 and C18:2 cis-9 cis-12 proportions in addition to the C18:3 cis-9 cis-12 cis-15 proportion (*p* < 0.05) up to 6% of supplementation when we were supplementing linseed oil in either its raw form (LO) or its nanoemulsified form (NLO). This was found where LO was added; a quadratic reduction (*p* < 0.05) was seen in the proportions of C18:1 cis-9, C18:2 cis-9 cis-12, and C18:3 cis-9 cis-12 cis-15. The proportion of SFA showed a quadratic increase (*p* < 0.001) when the LO levels of the addition were also considered. Increasing levels of the NLO addition resulted in a quadratic reduction of the SFA proportion, with a significance level of less than 0.05. In general, adding any amount of NLO led to a quadratic reduction (*p* < 0.001) in the proportions of the SFA and a quadratic elevation (*p* < 0.001) in the proportions of the PUFA in the fermentation fluid samples; both results were statistically significant. When compared to LO, the levels of volatile fatty acids, specifically acetate and propionate, were found to be significantly increased (*p* < 0.001) by NLO when supplemented with up to 6% of the DM. The same observations were also applicable in the case of volatile fatty acids.

Nanoemulsified oils reduced UFA to a lesser extent, revealing a more significant proportion (*p* < 0.01) of UFA, particularly PUFA, when compared to the proportion of SFA, which decreased (*p* < 0.01) by all nanoemulsified oil types. However, supplementing the raw form of oils at 3% resulted in a similar impact on the rumen fermentation parameters and fatty acid formation and accumulation as in nanoemulsified oils. That is why the second stage of trials targets tracking the effects of raw form and nanoemulsified form of olive oil, corn oil, and linseed oil supplemented at 3% of the DM compared to each other on the rumen fermentation pattern and selected microbial population.

### 3.3. Second Stage of Trials

Table 7 presents the results of research that looked at how adding raw and nanoemulsified olive oil, corn oil, and linseed oil to the diet affected the basic parameters of the rumen, as well as the volatile fatty acid and fatty acid composition of the animals. Compared to the diet that served as the control, the treatments that utilized nanoemulsified oils did not affect the pH of the fermentation culture or the ammonia concentration. However, the addition of NCO to the fermentation culture had a significantly more significant influence (*p* < 0.01) on the amount of ammonia, IVDMD, and total volatile fatty acid content compared to any raw oil supplements and the diet that served as the control. When nanoemulsion was added at a concentration of 3% DM, the molar proportions of both acetate and propionate were significantly increased. The addition of nanoemulsified oils to the fermentation culture at a concentration of 3% of DM led to a decrease (*p* < 0.001) in the proportion of vaccenic acid (trans-11 C18:1), as well as all CLA isomers. This was especially true for C18:2 cis-9 trans-11, which significantly decreased after NOO was added to the culture. In terms of C18 UFA, the supplementation of the nanoemulsified oils led to a significant rise (*p* < 0.01) in the proportions of oleic acid (cis-9 C18:1), linoleic acid (cis-9 cis-12 C18:2), and linolenic acid (cis-9 cis-12 cis-15 C18:3) when compared to the raw form and the control group. This was observed in all three. Table 8 presents the findings obtained from the quantitative PCR analysis on selected ruminal microbial populations. Compared to the other types of nanoemulsions, the raw form of oils, and the control treatment, it was found that the addition of NCO had a positive effect (*p* < 0.05) on the relative proportions of *Butyrivibrio fibrisolvens*, *Butyrivibrio proteoclasticus*, and *Ruminococcus albus*. In general, the addition of nanoemulsions increased (*p* < 0.05) the relative proportions of *Anaerovibrio lipolytica*, *Fibrobacter succinogenes*, and *Streptococcus bovis*, and this was especially true when NCO was added. Except for *Streptococcus bovis*, NLO was found to significantly decrease (*p* < 0.05) the relative proportion of most bacterial populations.

## 4. Discussion

Compared to the other treatments, the raw forms of oil blends rich in C18 unsaturated fatty acids (UFAs) included in both batch experiments caused a shift in the fatty acid proportion. These results demonstrated conclusively that rumen biohydrogenation in the batch cultures mirrored the rumen biohydrogenation under normal conditions. Previous research [27] has shown an increase in vaccenic acid (VA) and conjugated linoleic acid outflow from the rumen when dairy cows are fed C18 UFA-rich oils. Increasing the proportion of cis-9 trans-11 C18:2 in the rumen and ruminant products is, at this time, primarily constrained by the amount of vaccenic acid expelled from the rumen. Adding fish oil to the diets of ruminants is typically an effective way to accumulate VA. This efficiency is correlated with long-chain fatty acids, which may inhibit the final step in the biohydrogenation of C18:1 to C18:0 [28,29,30]. Khiaosa-ard et al. [28] performed lipid emulsification to improve the distribution of fatty acids in vitro biohydrogenation studies. They demonstrated that dispersing linoleic acid in an ultrasonic bath for only three minutes can significantly alter the proportion of fatty acids produced. They hypothesized that the small fatty acid droplets formed in stable emulsions by sonication tended to remain in the liquid phase rather than attaching to feed particles, thereby reducing the incidence of lipolysis and biohydrogenation in the fermentation fluid. In our case, however, the objectives were different; initially, we sought to disperse oils rich in polyunsaturated fatty acids in drinking water to increase the likelihood of using this supplement by dairy ruminants. In our preliminary experiments [14,15], we added the prepared nanoemulsified oil mixture directly to the incubation fluid instead of the feed. This was performed to simulate our original plan to add nanoemulsion to water.

In our experiment, nanoscale droplets of all proposed oils had a negative impact on the proportion of biohydrogenation intermediates (vaccenic acid and conjugated linoleic acid). Still, they did not affect the proportions of biohydrogenation (BH) bacteria. Based on the findings of [28], it is likely that the nanoscale diameter of the oil droplets, especially when supplemented at 3% and 6%, inhibited the chemical actions of ruminal bacteria without affecting their cellular structure. Our findings also indicated that the bacterial mortality rate is linearly affected by increasing nanoemulsion levels. Despite evidence indicating that ruminal biohydrogenation is a complex biochemical process involving a wide variety of fatty acid intermediates, the published pathways for linoleic acid (LA) BH have evolved over the years. A delay in the biohydrogenation of LA relative to alpha-linolenic acid (ALA) has been demonstrated; this may be due to the preferential uptake of ALA by rumen bacteria or to differences in the isomerase or saturase affinity of microorganisms between LA and ALA [29,30,31]. In addition, the rate of fatty acid release can vary significantly based on the source and structure of the added lipids [31,32,33,34,35].

Regarding nanoemulsion, it appears that this technology aids in retaining more significant proportions of polyunsaturated fatty acid than the raw addition. This observation may be due to the direct inhibition of ruminal lipolysis and/or biohydrogenation, which prevents a large proportion of PUFA from becoming saturated under biohydrogenation conditions; however, our results do not support this hypothesis. Nevertheless, according to Bauchart et al. [29], two distinct metabolic activities of biohydrogenation bacteria toward UFA, particularly linoleic acid, should be highlighted: first, the extensive biohydrogenation of UFA, and second, the protection of these UFA from biohydrogenation by the uptake and incorporation into cellular free fatty acids. Due to the nanodroplet size of the nanoemulsified form of used oil, the permeability or uptake of this fatty acid by the bacterial cell, and, consequently, the preservation of higher proportions of UFA from being biohydrogenated to SFA could be increased. This was supported by the subsequent studies and was confirmed by our in vivo finding regarding feeding nanoemulsified corn oil to lactating dairy goats [17].

## 5. Conclusions

In conclusion, the favorable increase in the in vitro rumen unsaturated fatty acids, particularly n-3 and n-6, that was observed when nanoemulsified oil forms were supplemented suggests that the nanoscale droplet of supplemented polyunsaturated fatty acids could be very effective in preserving higher proportions of polyunsaturated fatty acids, which will consequently be available for absorption regardless of the type of oil that was used. This was determined by observing the favorable increase in rumen unsaturated fatty acids. This discovery lends credence to the hypothesis that the nanoscale droplet of oil-in-water nanoemulsion may constitute a method that is both promising and capable of effectively supplementing unsaturated lipids. For instance, it is possible that an oil-in-water nanoemulsion may be mixed with clean drinking water and given to dairy cows to consume. However, additional research is still required, particularly at the molecular level, to establish the mechanism by which nanoscale oil droplets affect the bacteria in the rumen. This can only be accomplished by careful observation and analysis.

## Figures and Tables

**Table 1:** Ingredients and chemical composition of the control diet.

Item	Control Substrate
Ingredients, g/kg of DM	
Corn grain	75.5
Cotton seed meal	116
Sunflower seed meal	85.5
Wheat bran	175
Molasse	35.5
Mineral–vitamin mixture	12.5
Berseem clover	500
Chemical composition, g/kg of DM	
Organic Matter	911
Ash	89.0
Crude Protein	169
Either Extract	34.0
Neutral detergent fiber	349
Acid detergent fiber	199

**Table 2:** Fatty acid composition (g/100 g of FA) of the control substrate and supplements.

Item	Control Substrate	Supplements ^1^					
		OO	NOO	CO	NCO	LO	NLO
C14:0	0.74	0.02	0.02	0.08	0.12	0.10	0.09
C16:0	21.6	16.6	15.7	13.4	10.2	5.20	4.98
C18:0	2.89	2.70	2.49	2.01	1.82	3.80	3.52
C18:1 *cis*-9	21.8	61.2	62.9	29.7	31.2	18.7	19.3
C18:2 *cis*-9, *cis*-12	44.9	16.5	15.5	52.6	53.9	16.2	16.9
C18:3 cis-9, cis-12, cis-15	7.71	0.66	0.78	0.88	0.84	55.2	53.7
Other FA ^2^	0.36	2.32	2.61	1.33	1.92	0.80	1.51
SFA ^3^	25.6	19.8	18.8	15.5	12.1	9.50	9.20
UFA ^4^	74.7	80.2	81.2	84.5	87.9	90.5	90.8
MUFA ^5^	52.6	63.1	64.9	29.7	31.2	19.0	19.8
PUFA ^6^	21.8	17.2	16.3	54.8	56.7	71.5	71.0

^1^ Supplements: OO, olive oil; NOO, nanoemulsified olive oil; CO, corn oil; NCO, nanoemulsified corn oil. ^2^ Sum of other fatty acids, including C6:0, C8:0, C10:1, C11:0, C12:0, C13:0, C19:0, C18:2 *cis*-9 *cis*-15, C21:0, C20:2, and C22:0. ^3^ Sum of saturated fatty acids. ^4^ Sum of unsaturated fatty acids. ^5^ Sum of monounsaturated fatty acids. ^6^ Sum of polyunsaturated fatty acids.

**Table 3:** Forward (F) and reverse (R) primers used in the RT-PCR analysis of rumen bacteria.

Targeted Rumen Bacteria	Primer Sequence (5′ to 3′)	Reference
*Anaerovibrio lipolytica*	F: GAAATGGATTCTAGTGGCAAACG R: ACATCGGTCATGCGACCAA	[20]
*Butyrivibrio fibrisolvens*	F: ACACACCGCCCGTCACA R: TCCTTACGGTTGGGTCACAGA	[21]
*Butyrivibrio proteoclasticus*	F: TCCTAGTGTAGCGGTGAAATG R: TTAGCGACGGCACTGAATGCCTA	[22]
*Fibrobacter succinogenes*	F: GTTCGGAATTACTGGGCGTAAA R: CGCCTGCCCCTGAACTATC	[23]
*Megasphaera elsdenii*	F: AGATGGGGACAACAGCTGGA R: CGAAAGCTCCGAAGAGCCT	[24]
*Ruminococcus albus*	F: CCCTAAAAGCAGTCTTAGTTCG R: CCTCCTTGCGGTTAGAACA	[25]
*Ruminococcus flavefaciens*	F: CGAACGGAGATAATTTGAGTTTACTTAGG R: CGGTCTCTGTATGTTATGAGGTATTACC	[26]
*Streptococcus bovis*	F: TTCCTAGAGATAGGAAGTTTCTTCGG R: ATGATGGCAACTAACAATAGGGGT	[24]

**Table 4:** Effect of the supplementation of raw and nanoemulsified olive oil on basic rumen parameters, volatile fatty acid, and fatty acid composition.

Item	Form ^1^	Levels ^2^, %				SEM	Contrast ^3^		*p*-Value ^4^		
		0	3	6	9		L	Q	F	L	FxL
Rumen Basic Parameters											
pH	NOO	6.35 ^a^	6.32 ^a^	6.11 ^bx^	5.98 ^bx^	0.066	<0.001	<0.001	<0.001	0.002	0.123
	OO	6.35 ^a^	6.28 ^b^	5.69 ^cy^	5.23 ^dy^		0.001	0.009			
TGP, mL/g DM	NOO	264 ^d^	275 ^cx^	286 ^bx^	298 ^ax^	2.745	<0.001	0.002	0.004	0.015	0.214
	OO	264 ^a^	269 ^ay^	253 ^by^	248 ^cy^		<0.001	<0.001			
Ammonia-N, mmol/L	NOO	12.3 ^a^	12.6 ^a^	12.5 ^ax^	10.9 ^bx^	0.189	<0.001	0.011	<0.001	0.002	0.041
	OO	12.3 ^a^	12.1 ^a^	10.6 ^by^	9.47 ^cy^		<0.001	<0.001			
IVDMD ^5^, %	NOO	55.3 ^a^	56.2 ^ax^	54.8 ^ax^	49.6 ^bx^	0.832	0.004	0.019	0.003	0.012	0.110
	OO	55.3 ^a^	54.6 ^ay^	48.9 ^by^	41.7 ^cy^		<0.001	0.006			
Volatile Fatty Acids (VFA), mmol/L											
Total VFA	NOO	114 ^b^	120 ^ax^	119 ^ax^	101 ^cx^	1.877	<0.001	<0.001	0.002	0.022	0.236
	OO	114 ^a^	111 ^ay^	98.7 ^by^	87.7 ^cy^		<0.001	<0.001			
Acetate (A)	NOO	61.7 ^b^	67.3 ^ax^	60.9 ^bx^	53.7 ^cx^	1.286	<0.001	0.002	<0.001	0.009	0.098
	OO	61.7 ^a^	62.1 ^ay^	54.1 ^by^	42.3 ^cy^		0.003	0.009			
Propionate (P)	NOO	21.8 ^a^	22.3 ^ax^	20.9 ^bx^	19.3 ^cx^	0.279	<0.001	0.015	<0.001	<0.001	0.114
	OO	21.8 ^a^	21.1 ^ay^	19.2 ^by^	17.4 ^cy^		0.004	0.007			
Butyrate	NOO	19.6 ^a^	19.5 ^ax^	18.9 ^bx^	16.2 ^cx^	0.348	<0.001	0.018	<0.001	0.012	0.074
	OO	19.6 ^a^	18.7 ^by^	16.9 ^cy^	13.8 ^dy^		<0.001	<0.001			
A:P ratio	NOO	2.83 ^b^	3.02 ^a^	2.91 ^a^	2.78 ^cx^	0.029	<0.001	0.007	0.011	0.031	0.099
	OO	2.83 ^b^	2.94 ^a^	2.82 ^b^	2.43 ^cy^		0.008	0.009			
Fatty Acid Methyl Esters, g/100 g FA											
C14:0	NOO	2.89 ^a^	1.78 ^by^	1.58 ^cy^	1.63 ^cy^	0.095	<0.001	<0.001	0.002	0.009	0.127
	OO	2.89 ^a^	2.54 ^bx^	2.22 ^cx^	2.82 ^ax^		<0.001	<0.001			
C14:1 *cis*-9	NOO	2.12 ^a^	1.43 ^by^	1.11 ^cy^	1.07 ^cy^	0.066	<0.001	0.014	0.005	0.019	0.178
	OO	2.12 ^a^	1.64 ^bx^	1.56 ^cx^	1.44 ^dx^		<0.001	0.009			
C16:0	NOO	21.3 ^a^	18.7 ^by^	16.9 ^cy^	15.4 ^dy^	0.364	<0.001	<0.001	0.002	<0.001	0.022
	OO	21.3 ^a^	21.1 ^ax^	20.3 ^ax^	19.5 ^bx^		<0.001	<0.001			
C16:1 *cis*-9	NOO	0.67 ^c^	0.78 ^b^	0.83 ^bx^	0.89 ^ax^	0.014	0.008	0.017	<0.001	0.004	0.415
	OO	0.67 ^b^	0.81 ^a^	0.72 ^by^	0.69 ^by^		0.003	0.031			
C18:0	NOO	32.6 ^a^	26.7 ^by^	22.3 ^dy^	24.9 ^cy^	0.778	<0.001	<0.001	<0.001	<0.001	0.211
	OO	32.6 ^b^	33.9 ^abx^	34.7 ^ax^	26.8 ^cx^		<0.001	0.017			
C18:1 *trans*-10	NOO	0.89 ^ab^	0.81 ^by^	0.84 ^by^	0.92 ^ax^	0.016	<0.001	0.002	<0.001	0.004	0.166
	OO	0.89 ^b^	0.92 ^bx^	1.12 ^ax^	0.82 ^cy^		<0.001	0.001			
C18:1 *trans*-11	NOO	4.32 ^a^	3.85 ^by^	3.52 ^cy^	3.11 ^dy^	0.158	<0.001	<0.001	<0.001	0.003	0.147
	OO	4.32 ^d^	5.22 ^bx^	6.11 ^ax^	4.56 ^cx^		<0.001	<0.001			
C18:1 *cis*-9	NOO	5.66 ^d^	20.8 ^cx^	24.1 ^bx^	29.8 ^ax^	1.444	<0.001	<0.001	<0.001	<0.001	0.087
	OO	5.66 ^d^	11.3 ^cy^	12.8 ^by^	13.7 ^ay^		<0.001	0.004			
C18:2 *cis*-9 *cis*-12	NOO	3.09 ^d^	7.19 ^cx^	8.33 ^bx^	8.79 ^ax^	0.386	<0.001	<0.001	0.002	<0.001	0.009
	OO	3.09 ^d^	3.99 ^cy^	4.22 ^by^	4.78 ^ay^		<0.001	<0.001			
C18:2 *cis*-9 *trans*-11	NOO	0.26 ^b^	0.29 ^aby^	0.31 ^ay^	0.27 ^by^	0.016	<0.001	0.006	0.002	0.008	0.097
	OO	0.26 ^c^	0.43 ^bx^	0.52 ^ax^	0.41 ^bx^		0.002	0.011			
C18:2 *trans*-10 *cis*-12	NOO	0.19 ^a^	0.15 ^cy^	0.15 ^cy^	0.17 ^by^	0.017	0.011	0.079	<0.001	0.007	0.124
	OO	0.19 ^d^	0.29 ^cx^	0.35 ^bx^	0.42 ^ax^		<0.001	0.002			
C18:3 *cis*-9 *cis*-12 *cis*-15	NOO	0.48 ^c^	1.87 ^ax^	1.66 ^bx^	1.66 ^bx^	0.092	<0.001	<0.001	<0.001	<0.001	0.112
	OO	0.48 ^c^	0.82 ^by^	0.93 ^ay^	0.89 ^ay^		<0.001	0.006			
Other FA ^6^	NOO	25.5 ^a^	15.6 ^cy^	18.4 ^bx^	11.4 ^dy^	0.879	<0.001	0.011	0.007	0.003	0.072
	OO	25.5 ^a^	17.0 ^cx^	14.4 ^dy^	23.2 ^bx^		0.017	0.024			
SFA ^7^	NOO	69.4 ^a^	48.3 ^by^	44.4 ^cy^	42.3 ^dy^	1.793	<0.001	<0.001	<0.001	0.009	0.112
	OO	69.4 ^a^	63.2 ^bx^	60.7 ^cx^	59.2 ^cx^		<0.001	<0.001			
UFA ^8^	NOO	30.6 ^d^	51.7 ^cx^	55.6 ^bx^	57.7 ^ax^	1.393	<0.001	<0.001	<0.001	<0.001	0.278
	OO	30.6 ^d^	36.8 ^cy^	39.3 ^by^	40.8 ^ay^		<0.001	0.004			
MUFA ^9^	NOO	24.7 ^d^	33.8 ^cx^	35.9 ^bx^	37 ^ax^	0.804	<0.001	<0.001	0.002	0.001	0.114
	OO	24.7 ^c^	26.9 ^by^	29.2 ^ay^	30.3 ^ay^		<0.001	0.003			
PUFA ^10^	NOO	5.89 ^d^	17.9 ^cx^	19.7 ^bx^	20.7 ^ax^	0.998	<0.001	<0.001	<0.001	<0.001	0.088
	OO	5.89 ^c^	9.88 ^by^	10.1 ^aby^	10.4 ^ay^		<0.001	0.002			

^a–d^ Means within a row with different superscripts differ (*p* < 0.05). ^x,y^ Means within a column with different superscripts differ (*p* < 0.05). ^1^ Form of supplements, nanoemulsified olive oil (NOO) and raw olive oil (OO). ^2^ Levels of supplementation (%). ^3^ Contrast: significance of linear (L) and quadratic (Q) components of the response to the supplemented levels of oil in both nanoemulsified and raw forms. ^4^ The *p*-value: probability of significant effect due to oil form (F), level (L), and their interaction (F × L). ^5^ In vitro dry matter degradation. ^6^ Sum of other fatty acids, including C6, C10:1, C11:0, C16:1 *trans*, C18:1 *trans*-5, C18:1 *trans*-9, C18:1 *cis*-11, C18:1 *cis*-12, C18:1 *cis*-14, C18:1 *cis*-15, C18:2 *cis*-9 *cis*-15, C19:0, C20:1 *trans*, C21:0, C20:2, C22:0, C23:0, C22:2, C24:0, and C24:1. ^7^ Sum of saturated fatty acids. ^8^ Sum of unsaturated fatty acids. ^9^ Sum of monounsaturated fatty acids. ^10^ Sum of polyunsaturated fatty acids.

**Table 5:** Effect of the supplementation of raw and nanoemulsified corn oil on basic rumen parameters, volatile fatty acid, and fatty acid composition.

Item	Form ^1^	Levels ^2^, %				SEM	Contrast ^3^		*p*-Value ^4^		
		0	3	6	9		L	Q	F	L	FxL
Rumen Basic Parameters											
pH	NCO	6.55 ^a^	6.54 ^ax^	6.55 ^ax^	5.98 ^bx^	0.066	<0.001	0.022	<0.001	<0.001	0.411
	CO	6.55 ^a^	6.34 ^by^	5.87 ^cy^	5.33 ^dy^		<0.001	0.009			
TGP, mL/g DM	NCO	274 ^c^	290 ^bx^	301 ^ax^	309 ^ax^	3.259	<0.001	0.005	0.004	0.008	0.088
	CO	274 ^a^	272 ^ay^	261 ^by^	243 ^cy^		<0.001	0.007			
Ammonia-N, mmol/L	NCO	13.7 ^a^	13.7 ^a^	13.9 ^ax^	12.8 ^bx^	0.236	<0.001	0.004	<0.001	0.003	0.336
	CO	13.7 ^a^	13.5 ^a^	12.6 ^by^	9.22 ^cy^		<0.001	0.003			
IVDMD ^5^, %	NCO	56.6 ^a^	56.9 ^a^	57.3 ^ax^	50.6 ^bx^	0.940	<0.001	0.003	<0.001	<0.001	0.250
	CO	56.6 ^a^	56.7 ^a^	49.2 ^by^	39.8 ^cy^		<0.001	<0.001			
Volatile Fatty Acids (VFA), mmol/L											
Total VFA	NCO	122 ^b^	130 ^ax^	128 ^abx^	110 ^cx^	2.293	<0.001	0.022	<0.001	<0.001	0.009
	CO	122 ^a^	124 ^ay^	111 ^by^	83.5 ^cy^		<0.001	<0.001			
Acetate (A)	NCO	65.3 ^a^	66.2 ^a^	64.9 ^ax^	60.1 ^bx^	0.884	<0.001	0.002	<0.001	<0.001	0.412
	CO	65.3 ^a^	65.2 ^a^	61.1 ^by^	48.8 ^cy^		0.003	0.031			
Propionate (P)	NCO	24.7 ^a^	25.1 ^a^	24.8 ^ax^	20.2 ^bx^	0.489	<0.001	0.002	<0.001	<0.001	0.122
	CO	24.7 ^a^	24.9 ^a^	21.7 ^by^	16.2 ^cy^		<0.001	<0.001			
Butyrate	NCO	19.1 ^a^	19.5 ^ax^	19.5 ^ax^	17.1 ^bx^	0.283	<0.001	0.003	<0.001	0.004	0.369
	CO	19.1 ^a^	18.9 ^ay^	17.3 ^by^	14.1 ^cy^		<0.001	<0.001			
A:P ratio	NCO	2.64 ^b^	2.64 ^b^	2.62 ^by^	2.98 ^a^	0.025	0.005	0.019	<0.001	0.004	0.258
	CO	2.64 ^c^	2.62 ^c^	2.82 ^bx^	3.01 ^a^		0.012	0.029			
Fatty Acid Methyl Esters, g/100 g FA											
C14:0	NCO	3.32 ^a^	2.43 ^by^	2.12 ^cy^	1.87 ^d^	0.087	0.002	0.011	<0.001	0.005	0.149
	CO	3.32 ^a^	2.55 ^bx^	2.28 ^cx^	1.92 ^d^		<0.001	0.009			
C14:1 cis-9	NCO	2.88 ^a^	1.67 ^by^	1.43 ^cy^	1.22 ^dy^	0.101	<0.001	0.008	0.011	<0.001	0.228
	CO	2.88 ^a^	1.83 ^bx^	1.53 ^cx^	1.33 ^dx^		0.002	0.021			
C16:0	NCO	22.3 ^a^	19.1 ^by^	17.6 ^cy^	14.9 ^dy^	0.383	<0.001	<0.001	<0.001	0.009	0.018
	CO	22.3 ^a^	20.2 ^bx^	19.3 ^cx^	17.4 ^dx^		<0.001	0.002			
C16:1 *cis*-9	NCO	0.77 ^c^	0.82 ^b^	0.91 ^a^	0.95 ^a^	0.010	0.003	0.014	0.002	<0.001	0.111
	CO	0.77 ^c^	0.83 ^b^	0.87 ^ab^	0.92 ^a^		0.004	0.032			
C18:0	NCO	33.2 ^a^	25.4 ^by^	24.2 ^cy^	23.3 ^dy^	0.609	<0.001	<0.001	0.011	0.005	0.142
	CO	33.2^a^	30.7 ^bx^	31.2 ^abx^	29.9 ^bx^		0.003	0.014			
C18:1 *trans*-10	NCO	0.79	0.83 ^y^	0.81 ^y^	0.78 ^y^	0.016	<0.001	0.001	0.002	0.013	0.122
	CO	0.79 ^c^	0.97 ^ax^	1.08 ^ax^	0.81 ^bx^		0.002	0.007			
C18:1 *trans*-11	NCO	5.14 ^a^	4.01 ^by^	3.12 ^cy^	2.89 ^dy^	0.176	<0.001	<0.001	0.001	0.007	0.189
	CO	5.14 ^b^	5.54 ^abx^	6.12 ^ax^	4.09 ^cx^		<0.001	0.003			
C18:1 *cis*-9	NCO	5.89 ^d^	16.7 ^cx^	18.3 ^bx^	20.1 ^ax^	0.868	<0.001	<0.001	0.002	0.003	0.322
	CO	5.89 ^d^	10.2 ^ay^	9.12 ^by^	8.13 ^cy^		<0.001	0.006			
C18:2 *cis*-9 *cis*-12	NCO	3.12 ^d^	10.19 ^cx^	11.33 ^bx^	13.79 ^ax^	0.589	<0.001	<0.001	0.001	0.005	0.002
	CO	3.12 ^c^	6.77 ^ay^	6.32 ^ay^	5.66 ^by^		<0.001	0.002			
C18:2 *cis*-9 *trans*-11	NCO	0.32 ^a^	0.18 ^cy^	0.19 ^cy^	0.22 ^by^	0.017	<0.001	0.004	0.004	0.012	0.211
	CO	0.32 ^c^	0.42 ^bx^	0.49 ^ax^	0.38 ^bx^		0.002	0.008			
C18:2 *trans*-10 *cis*-12	NCO	0.26 ^a^	0.17 ^by^	0.18 ^by^	0.18 ^by^	0.012	<0.001	<0.001	0.002	<0.001	0.078
	CO	0.26 ^c^	0.31 ^bx^	0.39 ^ax^	0.32 ^bx^		0.002	0.008			
C18:3 *cis*-9 *cis*-12 *cis*-15	NCO	0.51 ^c^	1.97 ^ax^	1.89 ^ax^	1.79 ^bx^	0.091	<0.001	0.002	<0.001	0.003	0.125
	CO	0.51 ^b^	0.97 ^ay^	0.99 ^ay^	1.02 ^ay^		0.012	0.112			
Other FA ^6^	NCO	21.5 ^a^	16.5 ^dy^	17.9 ^cy^	18.0 ^by^	0.550	<0.001	<0.001	<0.001	0.006	0.116
	CO	21.5 ^b^	18.7 ^cx^	20.3 ^bx^	28.1 ^ax^		<0.001	<0.001			
SFA ^7^	NCO	67.4 ^a^	49.7 ^by^	46.3 ^by^	42.7 ^cy^	1.409	<0.001	<0.001	0.018	0.013	0.021
	CO	67.4 ^a^	60.1 ^bx^	59.2 ^bx^	58.1 ^bx^		<0.001	0.011			
UFA ^8^	NCO	32.6 ^d^	50.3 ^cx^	53.7 ^bx^	57.3 ^ax^	1.106	<0.001	0.002	0.019	0.014	0.114
	CO	32.6 ^d^	39.9 ^cy^	40.8 ^by^	41.9 ^ay^		<0.001	0.019			
MUFA ^9^	NCO	26.4 ^d^	29.8 ^cx^	32.6 ^bx^	37.1 ^ax^	0.539	<0.001	0.001	<0.001	0.002	0.412
	CO	26.4 ^d^	28.4 ^cy^	29.9 ^by^	31.8 ^ay^		<0.001	0.009			
PUFA ^10^	NCO	6.24 ^b^	20.5 ^ax^	21.1 ^ax^	20.2 ^ax^	0.960	<0.001	0.006	<0.001	<0.001	0.041
	CO	6.24 ^d^	11.5 ^ay^	10.9 ^by^	10.1 ^cy^		<0.001	<0.001			

^a–d^ Means within a row with different superscripts differ (*p* < 0.05). ^x,y^ Means within a column with different superscripts differ (*p* < 0.05). ^1^ Form of supplements, nanoemulsified corn oil (NCO) and raw corn oil (CO). ^2^ Levels of supplementation (%). ^3^ Contrast: significance of linear (L) and quadratic (Q) components of the response to the supplemented levels of oil in both nanoemulsified and raw forms. ^4^ The *p*-value: probability of significant effect due to oil form (F), level (L), and their interaction (F × L). ^5^ In vitro dry matter degradation. ^6^ Sum of other fatty acids, including C6, C10:1, C11:0, C16:1 *trans*, C18:1 *trans*-5, C18:1 *trans*-9, C18:1 *cis*-11, C18:1 *cis*-12, C18:1 *cis*-14, C18:1 *cis*-15, C18:2 *cis*-9 *cis*-15, C19:0, C20:1 *trans*, C21:0, C20:2, C22:0, C23:0, C22:2, C24:0, and C24:1. ^7^ Sum of saturated fatty acids. ^8^ Sum of unsaturated fatty acids. ^9^ Sum of monounsaturated fatty acids. ^10^ Sum of polyunsaturated fatty acids.

**Table 6:** Effect of the supplementation of raw and nanoemulsified linseed oil on basic rumen parameters, volatile fatty acid, and fatty acid composition.

Item	Form ^1^	Levels ^2^, %				SEM	Contrast ^3^		*p*-Value ^4^		
		0	3	6	9		L	Q	F	L	FxL
Rumen Basic Parameters											
pH	NLO	6.26 ^a^	6.24 ^ax^	6.04 ^bx^	5.80 ^cx^	0.075	<0.001	<0.001	<0.001	<0.001	0.073
	LO	6.26 ^a^	6.12 ^by^	5.61 ^cy^	5.12 ^dy^		<0.001	<0.001			
TGP, mL/g DM	NLO	261 ^d^	274 ^cx^	285 ^bx^	294 ^ax^	3.425	<0.001	0.089	0.004	<0.001	0.222
	LO	261 ^a^	262 ^ay^	249 ^by^	238 ^cy^		<0.001	0.066			
Ammonia-N, mmol/L	NLO	12.6 ^a^	12.8 ^a^	12.8 ^ax^	11.5 ^bx^	0.239	0.008	0.344	<0.001	0.002	0.041
	LO	12.6 ^a^	12.4 ^a^	11.2 ^by^	9.06 ^cy^		0.013	<0.001			
IVDMD ^5^, %	NLO	54.3 ^a^	54.8 ^a^	54.4 ^ax^	48.6 ^bx^	1.015	<0.001	0.002	<0.001	0.006	0.534
	LO	54.3 ^a^	53.9 ^a^	47.6 ^by^	39.5 ^cy^		<0.001	<0.001			
Volatile Fatty Acids (VFA), mmol/L											
Total VFA	NLO	114 ^b^	121 ^ax^	120 ^ax^	102 ^cx^	2.373	<0.001	<0.001	0.009	0.001	0.166
	LO	114 ^a^	114 ^ay^	102 ^by^	83.0 ^cy^		<0.001	<0.001			
Acetate (A)	NLO	61.6 ^b^	64.7 ^ax^	61.0 ^bx^	55.2 ^cx^	1.221	<0.001	0.003	0.001	0.022	0.211
	LO	61.6 ^a^	61.7 ^ay^	55.9 ^by^	44.2 ^cy^		<0.001	0.007			
Propionate (P)	NLO	22.6 ^a^	23.0 ^a^	22.2 ^ax^	19.2 ^bx^	0.441	0.014	0.059	<0.001	0.008	0.088
	LO	22.6 ^a^	22.3 ^a^	19.^8 by^	16.3 ^cy^		0.003	<0.001			
Butyrate	NLO	18.8 ^a^	18.9 ^a^	18.6 ^ax^	16.2 ^bx^	0.357	<0.001	0.001	<0.001	0.002	0.356
	LO	18.8 ^a^	18.2 ^a^	16.6 ^by^	13.5 ^cy^		<0.001	0.002			
A:P ratio	NLO	2.73	2.82	2.75	2.88 ^x^	0.011	0.082	0.195	0.021	0.144	0.321
	LO	2.73	2.77	2.82	2.71 ^y^		0.122	0.221			
Fatty Acid Methyl Esters, g/100 g FA											
C14:0	NLO	3.01 ^a^	2.04 ^by^	1.79 ^cy^	1.70 ^cy^	0.093	<0.001	0.011	0.002	0.018	0.144
	LO	3.01 ^a^	2.47 ^bx^	2.18 ^cx^	2.30 ^bx^		<0.001	0.003			
C14:1 cis-9	NLO	2.43 ^a^	1.50 ^by^	1.23 ^cy^	1.11 ^cy^	0.095	0.007	0.009	0.007	0.011	0.175
	LO	2.43 ^a^	1.68 ^bx^	1.50 ^cx^	1.34 ^dx^		<0.001	<0.001			
C16:0	NLO	21.1 ^a^	18.3 ^by^	16.7 ^cy^	14.7 ^dy^	0.417	<0.001	0.004	0.004	0.007	0.022
	LO	21.1 ^a^	20.0 ^ax^	19.2 ^ax^	17.9 ^bx^		<0.001	<0.001			
C16:1 *cis*-9	NLO	0.70 ^c^	0.78 ^b^	0.84 ^abx^	0.89 ^ax^	0.012	<0.001	<0.001	<0.001	0.001	0.478
	LO	0.70 ^b^	0.80 ^a^	0.77 ^ay^	0.78 ^ay^		<0.001	<0.001			
C18:0	NLO	31.9 ^a^	25.3 ^by^	22.5 ^by^	23.4 ^by^	0.762	<0.001	<0.001	<0.001	0.008	0.544
	LO	31.9 ^a^	31.3 ^ax^	31.9 ^ax^	27.5 ^bx^		<0.001	<0.001			
C18:1 *trans*-10	NLO	0.81	0.80 ^y^	0.80 ^y^	0.82	0.018	0.111	0.217	0.007	0.023	0.345
	LO	0.81 ^c^	0.92 ^bx^	1.07 ^ax^	0.79 ^c^		0.002	0.067			
C18:1 *trans*-11	NLO	4.59 ^a^	3.81 ^by^	3.22 ^by^	2.91 ^cy^	0.188	<0.001	<0.001	0.001	0.012	0.236
	LO	4.59 ^b^	5.22 ^ax^	5.93 ^ax^	4.20 ^bx^		<0.001	<0.001			
C18:1 *cis*-9	NLO	5.60 ^c^	18.2 ^bx^	20.6 ^abx^	24.2 ^ax^	1.300	<0.001	<0.001	<0.001	0.002	0.611
	LO	5.60 ^b^	10.4 ^ay^	10.63 ^ay^	10.59 ^ay^		<0.001	<0.001			
C18:2 *cis*-9 *cis*-12	NLO	3.03 ^c^	8.43 ^bx^	9.54 ^abx^	10.9 ^ax^	0.558	0.004	0.009	<0.001	0.004	0.222
	LO	3.03 ^b^	5.22 ^ay^	5.11 ^ay^	5.06 ^ay^		<0.001	0.005			
C18:2 *cis*-9 *trans*-11	NLO	0.28	0.23 ^y^	0.24 ^y^	0.24 ^y^	0.018	0.199	0.254	0.001	0.002	0.411
	LO	0.28 ^c^	0.41 ^abx^	0.49 ^ax^	0.38 ^bx^		0.002	0.009			
C18:2 *trans*-10 *cis*-12	NLO	0.22 ^a^	0.16 ^by^	0.16 ^by^	0.17 ^by^	0.016	0.018	0.119	<0.001	0.002	0.225
	LO	0.22 ^b^	0.29 ^bx^	0.36 ^ax^	0.36 ^ax^		<0.001	<0.001			
C18:3 *cis*-9 *cis*-12 *cis*-15	NLO	0.48 ^c^	1.86 ^ax^	1.72 ^abx^	1.67 ^bx^	0.104	0.011	0.156	<0.001	0.006	0.105
	LO	0.48 ^b^	0.87 ^ay^	0.93 ^ay^	0.93 ^ay^		0.002	0.008			
Other FA ^6^	NLO	25.8 ^a^	18.6 ^cy^	20.6 ^bx^	17.3 ^cy^	0.731	0.008	0.017	<0.001	<0.001	0.022
	LO	25.8 ^b^	20.3 ^cx^	19.9 ^cy^	27.9 ^ax^		0.003	0.008			
SFA ^7^	NLO	63.2 ^a^	47.8 ^by^	42.3 ^cy^	40.1 ^dy^	1.688	<0.001	<0.001	<0.001	<0.001	0.333
	LO	63.2 ^a^	58.8 ^bx^	57.3 ^bx^	53.9 ^cx^		<0.001	<0.001			
UFA ^8^	NLO	36.8 ^d^	52.2 ^cx^	57.7 ^bx^	59.9 ^ax^	1.138	<0.001	<0.001	<0.001	<0.001	0.211
	LO	36.8 ^c^	41.2 ^by^	42.7 ^by^	46.1 ^ay^		<0.001	0.009			
MUFA ^9^	NLO	28.5 ^d^	32.9 ^cx^	36.8 ^bx^	38.7 ^ax^	0.701	<0.001	<0.001	<0.001	<0.001	0.009
	LO	28.5 ^c^	30.3 ^by^	31.5 ^by^	34.2 ^ay^		<0.001	<0.001			
PUFA ^10^	NLO	8.32 ^c^	19.3 ^bx^	20.9 ^abx^	21.2 ^ax^	1.036	<0.001	<0.001	<0.001	<0.001	0.568
	LO	8.32 ^c^	10.9 ^by^	11.2 ^aby^	11.9 ^ay^		<0.001	0.066			

^a–d^ Means within a row with different superscripts differ (*p* < 0.05). ^x,y^ Means within a column with different superscripts differ (*p* < 0.05). ^1^ Form of supplements, nanoemulsified linseed oil (NLO) and raw linseed oil (LO). ^2^ Levels of supplementation (%). ^3^ Contrast: significance of linear (L) and quadratic (Q) components of the response to the supplemented levels of oil in both nanoemulsified and raw forms. ^4^ The *p*-value: probability of significant effect due to oil form (F), level (L), and their interaction (F × L). ^5^ In vitro dry matter degradation. ^6^ Sum of other fatty acids, including C6, C10:1, C11:0, C16:1 *trans*, C18:1 *trans*-5, C18:1 *trans*-9, C18:1 *cis*-11, C18:1 *cis*-12, C18:1 *cis*-14, C18:1 *cis*-15, C18:2 *cis*-9 *cis*-15, C19:0, C20:1 *trans*, C21:0, C20:2, C22:0, C23:0, C22:2, C24:0, and C24:1. ^7^ Sum of saturated fatty acids. ^8^ Sum of unsaturated fatty acids. ^9^ Sum of monounsaturated fatty acids. ^10^ Sum of polyunsaturated fatty acids.

**Table 7:** Effect of the supplementation of raw and nanoemulsified olive oil, corn oil, and linseed oil on basic rumen parameters, volatile fatty acid, and fatty acid composition.

Item		Treatments ^1^							SEM	*p*-Value
		CON	OO3%	CO3%	LO3%	NOO3%	NCO3%	NLO3%		
			Rumen Basic Parameters							
pH		6.37 ^b^	6.28 ^c^	6.35 ^b^	6.25 ^c^	6.46 ^a^	6.46 ^a^	6.42 ^a^	0.020	0.002
TGP, mL/g DM		265 ^b^	254 ^c^	257 ^c^	249 ^d^	268 ^ab^	270 ^a^	264 ^b^	1.914	<0.001
Ammonia-N, mmol/L		11.3 ^b^	10.5 ^c^	11.4 ^ab^	10.9 ^c^	11.7 ^a^	11.8 ^a^	11.1 ^b^	0.112	0.006
IVDMD ^2^, %		56.3 ^b^	54.6 ^c^	55.3 ^bc^	53.5 ^d^	56.9 ^ab^	57.1 ^a^	56.6 ^ab^	0.329	0.002
			Volatile Fatty Acids (VFA), mmol/L							
Total VFA		105 ^b^	100 ^c^	106 ^b^	99 ^c^	110 ^a^	112 ^a^	108 ^ab^	1.479	0.001
Acetate (A)		62.8 ^b^	60.7 ^c^	63.1 ^b^	60.1 ^c^	65.2 ^a^	66.7 ^a^	63.2 ^b^	0.321	<0.001
Propionate (P)		23.2 ^ab^	21.1 ^c^	22.7 ^b^	21.5 ^c^	23.7 ^a^	23.8 ^a^	23.5 ^a^	0.188	0.016
Butyrate		18.9 ^b^	18.1 ^c^	18.7 ^b^	17.2 ^d^	19.3 ^a^	19.4 ^a^	19.2 ^a^	0.109	<0.001
A:P ratio		2.71 ^b^	2.88 ^a^	2.78 ^ab^	2.80 ^ab^	2.75 ^b^	2.80 ^ab^	2.69 ^c^	0.010	0.023
			Fatty Acid Methyl Esters, g/100 g FA							
C14:0		2.71 ^a^	1.89 ^b^	1.77 ^bc^	1.81 ^b^	1.64 ^c^	1.52 ^d^	1.66 ^c^	0.097	0.001
C14:1 *cis*-9		1.98 ^a^	1.11 ^bc^	0.98 ^c^	1.23 ^ab^	0.94 ^cd^	0.87 ^d^	0.99 ^c^	0.094	0.009
C16:0		20.9 ^a^	18.7 ^b^	18.3 ^bc^	19.2 ^b^	16.4 ^d^	15.9 ^d^	17.1 ^c^	0.428	0.003
C16:1 *cis*-9		0.93 ^bc^	0.68 ^d^	0.95 ^b^	0.87 ^c^	0.81 ^cd^	1.04 ^a^	0.97 ^b^	0.029	0.017
C18:0		29.6 ^b^	31.9 ^a^	31.4 ^ab^	32.3 ^a^	27.1 ^c^	25.8 ^d^	26.4 ^d^	0.678	0.001
C18:1 *trans*-10		1.69 ^c^	1.98 ^b^	2.13 ^a^	2.27 ^a^	1.09 ^d^	1.17 ^d^	1.25 ^e^	0.120	<0.001
C18:1 *trans*-11		4.46 ^c^	5.76 ^b^	6.12 ^a^	5.89 ^b^	3.18 ^e^	3.42 ^d^	3.54 ^d^	0.315	<0.001
C18:1 *cis*-9		6.22 ^e^	13.7 ^c^	12.2 ^c^	11.7 ^d^	25.4 ^a^	20.3 ^b^	19.3 ^b^	1.586	<0.001
C18:2 *cis*-9 *cis*-12		3.87 ^e^	3.92 ^de^	4.56 ^c^	4.06 ^d^	8.31 ^b^	10.15 ^a^	9.88 ^a^	0.718	0.002
C18:2 *cis*-9 *trans*-11		0.21 ^e^	0.39 ^c^	0.51 ^a^	0.47 ^b^	0.19 ^e^	0.28 ^d^	0.26 ^d^	0.031	0.006
C18:2 *trans*-10 *cis*-12		0.13 ^d^	0.28 ^b^	0.31 ^ab^	0.35 ^a^	0.14 ^d^	0.16 ^c^	0.15 ^c^	0.023	0.001
C18:3 *cis*-9 *cis*-12 *cis*-15		0.38 ^e^	0.39 ^e^	0.43 ^e^	0.51 ^d^	1.12 ^c^	1.65 ^b^	1.97 ^a^	0.163	<0.001
Other FA ^3^		26.9 ^a^	19.3 ^c^	20.3 ^b^	19.3 ^c^	13.7 ^e^	17.7 ^d^	16.5 ^d^	1.006	<0.001
SFA ^4^		61.2 ^a^	59.1 ^b^	59.4 ^b^	58.3 ^b^	49.1 ^c^	47.2 ^d^	48.3 ^c^	1.505	0.013
UFA ^5^		38.8 ^e^	40.9 ^d^	40.6 ^d^	41.7 ^c^	50.9 ^b^	52.8 ^a^	51.7 ^a^	1.505	0.001
MUFA ^6^		30.6 ^c^	30.6 ^c^	28.7 ^e^	29.4 ^d^	35.1 ^a^	33.6 ^b^	33.3 ^b^	0.588	<0.001
PUFA ^7^		8.21 ^e^	10.3 ^d^	11.9 ^c^	12.3 ^c^	15.8 ^b^	19.2 ^a^	18.4 ^a^	1.021	<0.001

^a–e^ Means within a row with different superscripts differ (*p* < 0.05). ^1^ Treatments, control diet (CON), raw olive oil supplementation at 3% of DM (OO3%), raw corn oil supplementation at 3% of DM (CO3%), raw linseed oil supplementation at 3% of DM (LO3%), nanoemulsified olive oil supplementation at 3% of DM (NOO3%), nanoemulsified corn oil supplementation at 3% of DM (NCO3%), and nanoemulsified linseed oil supplementation at 3% of DM (NLO3%). ^2^ In vitro dry matter degradation. ^3^ Sum of other fatty acid including C6, C10:1, C11:0, C16:1 *trans*, C18:1 *trans*-5, C18:1 *trans*-9, C18:1 *cis*-11, C18:1 *cis*-12, C18:1 *cis*-14, C18:1 *cis*-15, C18:2 *cis*-9 *cis*-15, C19:0, C20:1 *trans*, C21:0, C20:2, C22:0, C23:0, C22:2, C24:0, and C24:1. ^4^ Sum of saturated fatty acids. ^5^ Sum of unsaturated fatty acids. ^6^ Sum of monounsaturated fatty acids. ^7^ Sum of polyunsaturated fatty acids.

**Table 8:** Effect of the supplementation of raw and nanoemulsified olive oil, corn oil, and linseed oil on ruminal microbial populations quantified using quantitative PCR.

Item ^1^	Treatments ^2^							SEM	*p*-Value
	CON	OO3%	CO3%	LO3%	NOO3%	NCO3%	NLO3%		
*Anaerovibrio lipolytica*	1.01 ^b^	0.93 ^c^	0.98 ^c^	0.87 ^d^	1.08 ^a^	1.12 ^a^	1.03 ^b^	0.024	0.001
*Butyrivibrio fibrisolvens*	1.22 ^b^	1.17 ^c^	1.18 ^c^	1.15 ^d^	1.26 ^ab^	1.32 ^a^	1.21 ^b^	0.016	0.011
*Butyrivibrio proteoclasticus*	2.77 ^b^	2.71 ^c^	2.75 ^b^	2.69 ^c^	2.83 ^ab^	2.91 ^a^	2.79 ^b^	0.021	0.001
*Fibrobacter succinogenes*	3.56 ^c^	3.45 ^d^	3.52 ^c^	3.42 ^d^	3.76 ^a^	3.77 ^a^	3.68 ^b^	0.040	<0.001
*Megasphaera elsdenii*	0.49 ^b^	0.33 ^d^	0.37 ^c^	0.31 ^d^	0.49 ^b^	0.54 ^a^	0.48 ^b^	0.025	<0.001
*Ruminococcus flavefaciens*	0.63 ^b^	0.58 ^c^	0.61 ^bc^	0.57 ^c^	0.67 ^b^	0.71 ^a^	0.64 ^b^	0.014	0.003
*Ruminococcus albus*	0.056 ^ab^	0.051 ^bc^	0.054 ^b^	0.047 ^c^	0.055 ^ab^	0.059 ^a^	0.054 ^b^	0.001	0.009
*Streptococcus bovis*	0.028 ^a^	0.021 ^bc^	0.023 ^b^	0.019 ^c^	0.027 ^a^	0.031 ^a^	0.027 ^a^	0.021	0.010

^a–d^ Means within a row with different superscripts differ (*p* < 0.05). ^1^ Rumen bacteria expressed as an arbitrary unit. ^2^ Treatments, control diet (CON), raw olive oil supplementation at 3% of DM (OO3%), raw corn oil supplementation at 3% of DM (CO3%), raw linseed oil supplementation at 3% of DM (LO3%), nanoemulsified olive oil supplementation at 3% of DM (NOO3%), nanoemulsified corn oil supplementation at 3% of DM (NCO3%), and nanoemulsified linseed oil supplementation at 3% of DM (NLO3%).

## Data Availability

The data presented in this study are available upon request from the corresponding author.
